# Long-term Non-Invasive Ventilation in Infants: A Systematic Review and Meta-Analysis

**DOI:** 10.3389/fped.2018.00013

**Published:** 2018-02-12

**Authors:** Prabhjot K. Bedi, Maria Luisa Castro-Codesal, Robin Featherstone, Mohammed M. AlBalawi, Bashar Alkhaledi, Anita L. Kozyrskyj, Carlos Flores-Mir, Joanna E. MacLean

**Affiliations:** ^1^Department of Pediatrics, Faculty of Medicine and Dentistry, University of Alberta, Edmonton, AB, Canada; ^2^Alberta Research Centre for Health Evidence, University of Alberta, Knowledge Translation Platform, Alberta SPOR SUPPORT Unit, Edmonton, AB, Canada; ^3^Department of Pediatrics, King Faisal Specialist Hospital and Research Centre, Riyadh, Saudi Arabia; ^4^Pediatric Pulmonary and Sleep Unit, Al-Sabah Hospital, Kuwait City, Kuwait; ^5^Women and Children’s Health Research Institute, University of Alberta, Edmonton, AB, Canada; ^6^Faculty of Medicine and Dentistry, School of Dentistry, University of Alberta, Edmonton, AB, Canada

**Keywords:** continuous positive airway pressure, bi-level positive airway pressure, obstructive sleep apnea, Pierre Robin sequence, laryngo-tracheomalacia, spinal muscular atrophy type 1, central hypoventilation syndrome

## Abstract

**Background:**

The use of long-term non-invasive ventilation (NIV) to treat sleep and breathing disorders in children has increased substantially in the last decade; however, less data exist about its use in infants. Given that infants have distinct sleep and breathing patterns when compared to older children, the outcomes of infants on long-term NIV may differ as well. The aim of this study is to systematically review the use and outcomes of long-term NIV in infants.

**Methods:**

Ovid Medline, Ovid Embase, CINAHL (via EbscoHOST), PubMed, and Wiley Cochrane Library were systematically searched from January 1990 to July 2017. Studies on infants using long-term NIV outside of an acute care setting were included. Data were extracted on study design, population characteristics, and NIV outcomes.

**Results:**

A total of 327 studies were full-text reviewed, with final inclusion of 60. Studies were distributed across airway (40%), neuromuscular (28%), central nervous system (10%), cardio-respiratory (2%), and multiple (20%) disease categories. Of the 18 airway studies reporting on NIV outcomes, 13 (72%) reported improvements in respiratory parameters. Of the 12 neuromuscular studies exclusively on spinal muscular atrophy type 1 (SMA1), six (50%) reported decreased hospitalizations and nine (75%) reported on mortality outcomes. Risk of bias was moderate to serious, and quality of the evidence was low to very low for all studies. Most studies had an observational design with no control group, limiting the potential for a meta-analysis.

**Conclusion:**

The outcomes reported in studies differed by the disease category being studied. Studies on airway conditions showed improvements in respiratory parameters for infants using NIV. Studies on neuromuscular disorder, which were almost exclusively on SMA1, reported decreased hospitalizations and prolonged survival. Overall, it appears that NIV is an effective long-term therapy for infants. However, the high risk of bias and low quality of the available evidence limited strong conclusions.

## Introduction

### Rationale

Long-term non-invasive ventilation (NIV), defined as respiratory support delivered through an interface outside the airway, has become the treatment of choice for a number of chronic conditions resulting in respiratory insufficiency or sleep and breathing disorders in infants and children (1–3). These conditions include airway disorders, neuromuscular disorders (NMDs), and disorders of the central nervous system (CNS) (3–6). The shift toward NIV therapies may have been driven by improvements in NIV technology, a greater emphasis on home-based care, and a growing acceptance of NIV as a viable long-term respiratory support (1, 6, 7). With the increasing number of infants and children living at home using NIV, understanding the benefits and risks of NIV is becoming important not only for specialists involved in starting this therapy but also for pediatricians and primary care physicians providing care to these children within the community and policy makers responsible for decisions about provision of healthcare resources.

While there is a considerable body of work describing the use of long-term NIV, including continuous positive airway pressure (CPAP) and bi-level positive airway pressure (BPAP), in a broad range of pediatric populations, less is known about its use in infants (8–10). Without sufficient data to suggest otherwise, similar NIV treatment approaches are likely followed in both infants and older children, despite key physiological differences in sleep and breathing patterns in infancy. Both sleep and breathing processes are immature at birth and continue to develop through infancy, resulting in change in sleep patterns and breathing control that continue through early life (11). Sleep occupies a greater proportion of time in infants compared to older children (12), which makes infants more vulnerable to respiratory disorders that disrupt sleep. Immaturity of central respiratory centers in infants contributes to increased respiratory events and a greater variability in oxygen saturation, both of which may be important for the normal development of respiratory control (11, 13). Since sleep and breathing processes differ by age, especially in early life, the type of respiratory and sleep disorders treated with NIV, the response to NIV treatment, and the outcomes for NIV may also differ in infants as compared to older children.

Most data available on long-term NIV use in infants is limited to single-center observational studies with relatively small sample sizes (8). Aggregation of the available data for combined data analysis will improve our understanding of the risks and benefits of NIV therapy in the infant population.

### Objective

The objective of this systematic review is to summarize the available evidence on the use of long-term NIV for infants and to estimate effect sizes for specific sub-populations and clinical outcomes compared to alternative respiratory care strategies.

### Research Question

Does the use of NIV, compared to supportive care, or invasive ventilation, improve clinical outcomes for infants under the age of 2 years with chronic conditions resulting in respiratory insufficiency or sleep and breathing disorders?

## Methods

### Study Design

This review was conducted using systematic review methodology.

### Participants

The inclusion criteria for this systematic review were as follows: (1) infants, defined by the Public Health Agency of Canada as ages 0–24 months inclusive (14); (2) NIV use, defined as breathing support delivered from outside the airway; and (3) long-term NIV use, defined as greater than three months outside of an acute care setting. For studies that examined a broader age range, the mean age of NIV initiation had to be less than 24 months in order to be included in this review, or data had to be presented separately for infants. We did not place any restrictions on study design or outcome eligibility.

### Systematic Review Protocol

The protocol for this systematic review was developed according to the Preferred Reporting Items for Systematic Review and Meta-Analyses (PRISMA) guidelines (15). The full protocol has been registered in the PROSPERO database for international prospective reviews (16).

### Search Strategy

This systematic review is an extension of a prior scoping review on long-term NIV in children (8). The scoping review search strategy, using Medical Subject Headings (MeSH) and free-text terms for “child” and “non-invasive ventilation,” was developed for MEDLINE (Ovid) and adapted for subsequent electronic databases with the full protocol published elsewhere (17) [see Table 1 for original MEDLINE (Ovid) search strategy]. Human studies published from 1990 onward were searched in MEDLINE (Ovid), Embase (Ovid), CINAHL (Ebsco), Cochrane Library (Wiley), and PubMed between November 17 and 28, 2014, with no restriction on study design. Gray literature, in the form of conference abstracts on respiratory and sleep medicine, was identified from 2012 to 2014. The literature search was re-run on April 29, 2016, and July 12, 2017, using the same search strategy in Ovid MEDLINE, Ovid Embase, CINAHL, and Wiley Cochrane Library to identify additional studies.

**Table 1:** Search strategy used in the Ovid Medline database for the scoping review to identify literature on the use of long-term non-invasive ventilation in children.

Ovid MEDLINE(R) In-Process and other non-indexed citations and Ovid Medline(R): 1946 to November Week 1, 2014	

Original search date: 17 November 2014	

Update search dates: 29 April 2016 and 12 July 2017	
1. Continuous Positive Airway Pressure/ 2. Noninvasive Ventilation/ 3. Intermittent Positive-Pressure Breathing/ 4. Ventilators, Negative-Pressure/ 5. AVAPS.tw. 6. [(auto* or adaptive) adj2 (servoventilation or ventilation)].tw. 7. AutoSet*.tw. 8. ((bi level or bi-level) adj2 (airway* or air way* or assist* or breath* or positive pressure* or respirat* or ventilat* or support* or therap*)).tw. 9. BIPAP*.tw. 10. BPAP*.tw. 11. c flex.tw. 12. CNEP.tw. 13. (continuous negative adj2 pressure).tw. 14. (continuous positive airway* or continuous positive air way*).tw. 15. (continuous positive adj2 pressure).tw. 16. CPAP*.tw. 17. ((domicil* or home*) adj5 ventilat*).tw. 18. intermittent positive pressure breathing.tw. 19. IPPB*.tw. 20. ((long term or longterm) adj5 ventilat*).tw. 21. ((nasal* or mask*) adj2 (positive adj2 pressure)).tw. 22. ((nasal* or mask*) adj2 ventilat*).tw. 23. nCPAP*.tw. 24. ((negative pressure) adj2 (respirat* or ventilat*)).tw. 25. ((night* or nocturnal* or sleep*) adj5 ventilat*).tw. 26. NIPPV*.tw. 27. ((noninvasive adj5 ventilat*) or (non invasive adj5 ventilat*)).tw. 28. (noninvasive respiratory support* or non invasive respiratory support*).tw. 29. NPPV*.tw. 30. (positive pressure adj2 respirat*).tw. 31. REMstar*.tw. 32. (tank adj (respirat* or ventilat*)).tw. 33. VPAP*.tw. 34. or/1–33 35. Hypoventilation/pc, rh, th [Prevention & Control, Rehabilitation, Therapy] 36. Interactive Ventilatory Support/ 37. Intermittent Positive-Pressure Ventilation/ 38. Positive-Pressure Respiration/ 39. Respiration, Artificial/ 40. Respiratory Insufficiency/pc, rh, th [Prevention & Control, Rehabilitation, Therapy]	41. exp Sleep Apnea Syndromes/pc, rh, th [Prevention & Control, Rehabilitation, Therapy] 42. Ventilators, Mechanical/ 43. ((airway* or air way* or breath* or inspirat* or respirat* or ventilat*) and (positive adj2 pressure)).tw. 44. intermittent positive pressure.tw. 45. IPPV*.tw. 46. (mechanical adj (respirat* or ventilat*)).tw. 47. (positive adj2 pressure adj (assist* or support* or therap*)).tw. 48. positive airway pressure.tw. 49. pulmonary ventilator*.tw. 50. respiratory support*.tw. 51. or/35–50 52. (noninvasive or non invasive or spontaneous*).mp. 53. 51 and 52 54. 34 or 53 55. exp Adolescent/ 56. exp Child/ 57. exp Infant/ 58. exp Minors/ 59. exp Pediatrics/ 60. exp Puberty/ 61. exp Schools/ 62. adoles*.mp. 63. (baby* or babies or infant* or infancy or neonat* or newborn* or postmatur* or prematur* or preterm*).mp. 64. (boy* or girl* or teen*).mp. 65. (child* or kid or kids or preschool* or school age* or schoolchild* or toddler*).mp. 66. (elementary school* or high school* or highschool* or kindergar* or nursery school* or primary school* or secondary school*).mp. 67. minors*.mp. 68. (pediatric* or peadiatric* or pediatric*).mp. 69. (prepubescen* or pubescen* or pubert*).mp. 70. or/55–69 71. 54 and 70 72. (case reports or comment or editorial or letter).pt. 73. 71 not 72 74. exp animals/not humans.sh. 75. 73 not 74 76. limit 75 to yr = “1990-Current” 77. remove duplicates from 76

*The search strategy also included infant keywords to help identify studies on infants*.

### Data sources, Study Selection, and Data Extraction

The titles and abstracts of studies identified by the literature search were screened by two reviewers (JEM and MCC) to determine eligibility for full-text retrieval. English, French, Spanish, and Portuguese studies that were considered eligible were full-text reviewed for inclusion by two reviewers (JEM and MCC). The final included studies pertaining to children 0–18 years were then full-text screened by two reviewers (PKB and MMA) to identify studies relevant to infants for inclusion in this systematic review. Any disagreement at the screening, eligibility, and inclusion levels were discussed until a consensus was reached. The reference lists of studies meeting inclusion were also reviewed to identify any additional relevant literature.

Data were entered into a pre-established data collection form in Microsoft Excel (version 14.0.4760, Microsoft Corporation, 2010). These data included author’s name, year of publication, country of publication, study design, sample size, age of NIV initiation, NIV type, primary underlying disease conditions, co-morbidities, and primary and secondary outcome measures. One reviewer (PKB) extracted the data, and 20% of data extraction was verified by a second reviewer (MCC).

### Risk of Bias

The Cochrane Risk of Bias in Non-Randomized Studies of Interventions (ROBINS-I) tool (18) was used to assess the risk of bias in individual studies. The tool measured confounding, selection, measurement, missing data, and reporting bias. Bias was ranked as low, moderate, severe, critical, or no information. Risk of bias in individual studies was independently assessed by two reviewers (PKB and MMA), with disagreements resolved by discussion and consensus.

### Quality Assessment

The Grading of Recommendations Assessment, Development and Evaluation (GRADE) tool (19) was used to determine the quality of studies at an outcome level. Two reviewers (PKB and MMA) independently assessed the quality of studies, with disagreements being resolved through discussion and consensus. Meta-analysis was performed to calculate risk ratios for appropriate outcomes using Review Manager (version 5.3., Copenhagen: The Nordic Cochrane Centre, The Cochrane Collaboration, 2014).

### Synthesis of Results

Studies were grouped by disease category (airway, NMD, CNS, cardio-respiratory or multiple disorders) after the data collection stage, to allow for adequate pathophysiological comparisons. Within each disease category, studies were grouped based on primary disease conditions. We included studies with infants who had multiple disease conditions under one disease heading if >75% of the infant cohort had the same disease condition; otherwise these studies were included in the multiple disorders category.

Primary and secondary outcomes were established after data collection, during synthesis of the data, based on the most common and clinically relevant outcomes reported in studies with the same disease condition. Primary outcomes were as follows: (1) objective changes in respiratory parameters, (2) discontinuation of NIV, (3) hospitalizations, and (4) mortality. Secondary outcomes were as follows: (1) improvements in underlying disease conditions, (2) improvements in growth parameters, (3) NIV facilitation of extubation, (4) predictors of NIV requirement, (5) NIV success/failure, (6) adherence to respiratory support, and (7) mask complications. Studies were included in the synthesis if they reported on at least one primary or secondary outcome. Continuous data were presented as a weighted mean (standard deviation) or median (interquartile range) where appropriate. Results were grouped and reported based on the primary underlying disease category being studied. Primary outcomes were reported in both tabular and narrative format, while secondary outcomes were only reported narratively.

## Results

### Study Selection and Characteristics

**Figure 1:**
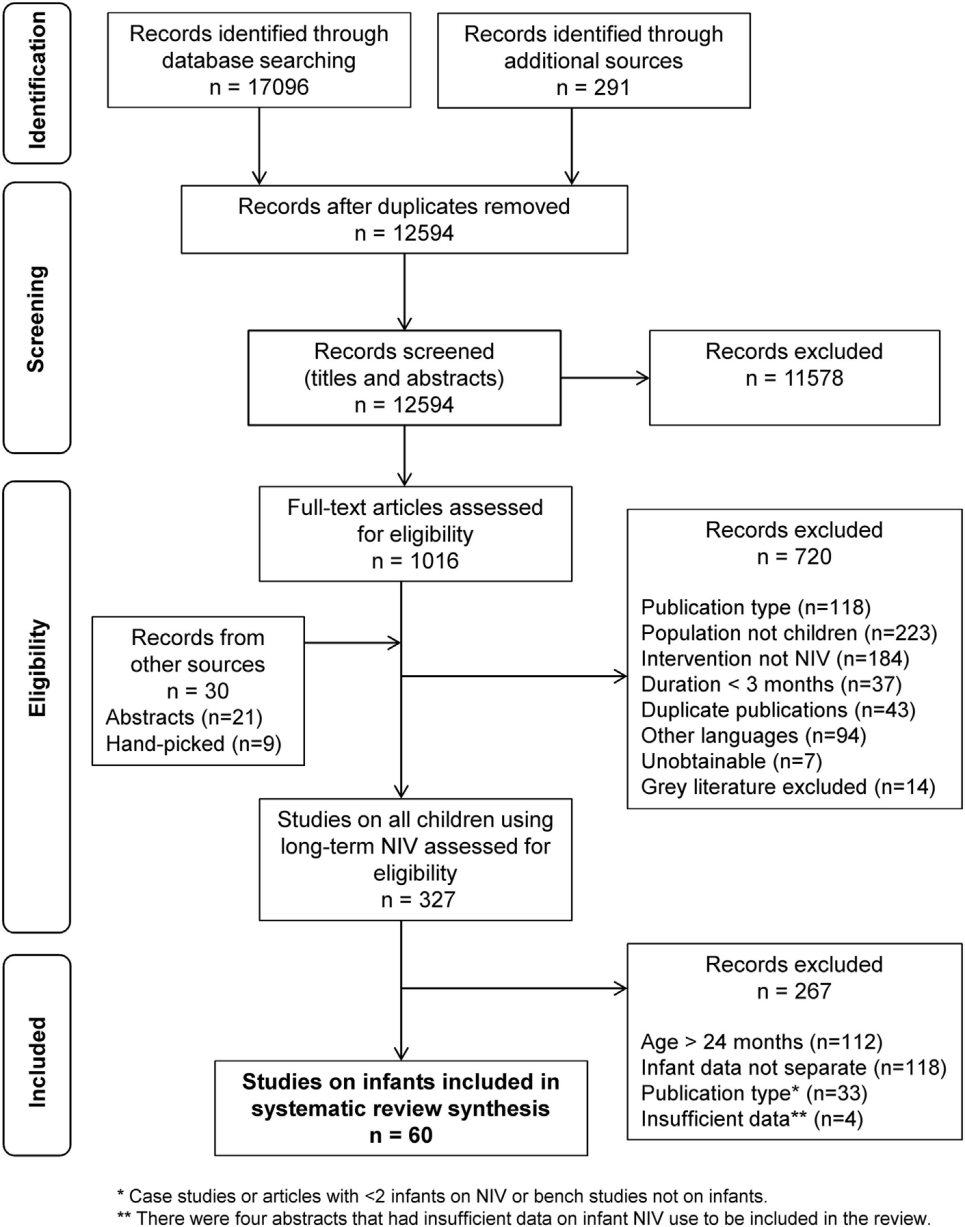
Flow diagram outlining the study selection process for the systematic review, following the Preferred Reporting Items for Systematic Reviews and Meta-Analyses guidelines (15).

The search strategy, after removal of duplicates, identified 12,594 studies and additional records (Figure 1). After screening of the titles and abstracts, and with the addition of records from additional sources, 1046 studies met eligibility for review. After full-text review, 327 studies on children ages 0–18 years met the inclusion criteria for the scoping review. Full-text review of these 327 articles identified 64 studies meeting the infant inclusion criteria. Four conference proceedings met inclusion criteria but were excluded because of insufficient data reporting, leaving 60 articles reporting on a total of 977 infants for inclusion in this systematic review (Table 2) (3, 7, 9, 10, 20–75).

**Table 2:** Characteristics and outcomes of 60 studies included in the systematic review on infants using long-term NIV.

First author, year, country	Study design	Study duration	Total *n*(M/F)	Infants on NIV	Age [mean ± SD or median (range) unless otherwise stated]	Interventions	Infant NIV outcomes	
							Primary	Secondary
**Articles on airway disorders: obstructive sleep apnea**								

Downey (20), 2000, USA	Quantitative: observational (cohort)	7 years	18 (n/a)	*n* = 10^a^	Overall: <2 years	CPAP (*n* = 14) IMV (*n* = 4)	• Changes in respiratory parameters • Discontinuation of NIV	• Number of subjects on NIV

Guilleminault (21), 1995, USA	Quantitative: observational (cohort)	n/a	74 (35/39)	*n* = 74	24 ± 9 weeks	CPAP (*n* = 74)	• Discontinuation of NIV	• Number of subjects on NIV

Harrington (22), 2003, Australia, Finland	Quantitative: observational (case–control)	n/a	18 (11/7)	*n* = 6	13 ± 4 weeks	CPAP (*n* = 6)	• Changes in respiratory parameters	• Number of subjects on NIV

Leonardis (23), 2013, USA	Quantitative: observational (cross-sectional)	4 years	126 (86/40)	*n* = 18	NIV group: 16 months	None (*n* = 33) NIV (*n* = 18) IMV (*n* = 7)^b^	• Changes in respiratory parameters	• Number of subjects on NIV

Liu (24), 2012, China	Quantitative: observational (case series)	n/a	3 (2/1)	*n* = 2	Overall: 1 month to 5 years Infants: 1–7 months	CPAP (*n* = 2) BPAP (*n* = 2)	• Changes in respiratory parameters	• Number of subjects on NIV • Benefit of NIV (growth parameters)

Marcus (25), 1995, USA	Quantitative: observational (cross-sectional)	n/a	94 (60/34)	*n* = 3^c^	Overall: <1–19 years Infants: <1 year (*n* = 3)	CPAP (*n* = 94)		• Number of subjects on NIV*

Massa (26), 2002, UK	Quantitative: observational (cohort)	5 years	66 (39/27)	*n* = 9^c^	Overall: 5.9 ± 5.1 years Infants: <1 year (*n* = 18)	CPAP (*n* = 66)		• Number of subjects on NIV*

McNamara (27), 1995, Australia	Quantitative: control before–after	0.5 years	5 (2/3)	*n* = 5	8–12 weeks	CPAP (*n* = 5)	• Changes in respiratory parameters • Discontinuation of NIV • Survival/mortality	• Number of subjects on NIV

McNamara (28), 1999, Australia	Quantitative: observational (case–control)	n/a	24 (13/11)	*n* = 8	CPAP group: 10.8 ± 1.3 weeks	CPAP (*n* = 8)	• Changes in respiratory parameters • Discontinuation of NIV	• Number of subjects on NIV

McNamara (29), 1999, Australia	Quantitative: observational (cohort)	n/a	24 (15/9)	*n* = 24	1–51 weeks	CPAP (*n* = 24)	• Changes in respiratory parameters	• Number of subjects on NIV

Robison (10), 2013, USA	Quantitative: observational (cross-sectional)	4 years	295 (196/99)	*n* = 18	CPAP/bi-level group: 15.6 months (3–29 months)	None (*n* = 76) NIV (*n* = 18) T&A (*n* = 116) IMV (*n* = 6)^b^	• Changes in respiratory parameters	• Number of subjects on NIV

Rosen (30), 2010, USA	Quantitative: observational (case series)	5.5 years	16 (n/a)	*n* = 6	Overall: <2 years	CPAP (*n* = 6)	• Discontinuation of NIV	• Number of subjects on NIV

**Articles on airway disorders: Pierre Robin sequence**								

Amaddeo (31), 2016, France	Quantitative: observational (cohort)	1 year	44 (n/a)	*n* = 9	Infants: 0–2 months	CPAP (*n* = 9)	• Changes in respiratory parameters • Discontinuation of NIV • Hospitalizations	• Number of subjects on NIVAdherence to NIV

Cheng (32), 2011, Australia	Quantitative: observational (case series)	5 years	6 (n/a)	*n* = 6	26 days to 11 months	CPAP (*n* = 6)		• Number of subjects on NIV*

Daniel (33), 2013, Australia	Quantitative: observational (cross-sectional)	12 years	39 (16/23)	*n* = 18	n/a	CPAP (*n* = 18)		• Number of subjects on NIV*

Goudy (34), 2017, USA	Quantitative: observational (cohort)	9 years	38 (18/20)	*n* = 9	n/a (neonates)	NIV (*n* = 9) NPA (*n* = 14) IMV (*n* = 8) MDO (*n* = 5)		• Number of subjects on NIV • NIV success/failure

Kam (35), 2015, Canada	Quantitative: observational (cohort)	11 years	139 (72/67)	*n* = 20^d^	23 months (5 days to 8 years)	None (*n* = 61) CPAP (*n* = 20) IMV (*n* = 19)^b^	• Hospitalizations	• Number of subjects on NIV

Leboulanger (36), 2010, France	Quantitative: observational (case series)	10 years	7 (3/4)	*n* = 7	1–10 months	CPAP (*n* = 5) BPAP (*n* = 2)	• Changes in respiratory parameters • Discontinuation of NIV	• Number of subjects on NIV • Adherence to NIV

Müller-Hagedorn (37), 2017, Germany	Quantitative: observational (cohort)	7 years	68 (n/a)	*n* = 5	n/a	CPAP (*n* = 5)		• Number of subjects on NIV*

**Articles on upper airway disorders: Laryngo-tracheomalacia**								

Essouri (38), 2005, France	Quantitative: control before–after	n/a	10 (5/5)	*n* = 10	9.5 months (3–18 months)	None (*n* = 10) CPAP (*n* = 10) BPAP (*n* = 10)	• Changes in respiratory parameters	• Number of subjects on NIV

Fauroux (39), 2001, France, UK	Quantitative: control before–after	n/a	12 (10/2)	*n* = 5	Overall: 32.9 ± 25.8 months Infants: 8–19 months	None (*n* = 12) BPAP (*n* = 12)	• Changes in respiratory parameters • Discontinuation of NIV	• Number of subjects on NIV • Adherence to NIV • Benefit of NIV (growth parameters)

Shatz (40), 2004, Israel	Quantitative: observational (cohort)	3 years	50 (36/14)	*n* = 50	6.5 ± 3.5 months (1–18 months)	CPAP (*n* = 5) BPAP (*n* = 9)	• Discontinuation of NIV	• Number of subjects on NIV • Improvement in underlying disease

Zwacka (41), 1997, Germany	Quantitative: observational (case series)	n/a	10 (5/5)	*n* = 10	3 weeks to 5 months	CPAP (*n* = 7)	• Changes in respiratory parameters	• Number of subjects on NIV • Benefit of NIV (growth parameters)

**Articles on airway disorders: breath holding spells**								

Guilleminault (42), 2007, USA, Taiwan	Quantitative: observational (case–control)	2.5 years	19 (11/8)	*n* = 14	31 ± 3 weeks	CPAP (*n* = 14)	• Changes in respiratory parameters	• Number of subjects on NIV • NIV success/failure

**Articles on neuromuscular disease: spinal muscular atrophy type 1**								

Bach (43), 2000, USA	Quantitative: observational (case series)	n/a	11 (6/5)	*n* = 8	3–28 months	BPAP (*n* = 11)	• Hospitalizations • Survival/Mortality	• Number of subjects on NIV • Benefit of NIV (extubation) • Benefit of NIV (growth parameters)

Bach (44), 2002, USA	Quantitative: observational (cohort)	5 years	56 (n/a)	*n* = 33	Overall for patient groups: NIV: 11.2 ± 5.7 months IMV: 10.8 ± 5.0months supportive: 6.0 ± 1.3 months	NIV (*n* = 33) IMV (*n* = 16) None (*n* = 7)	• Hospitalizations • Survival/mortality	• Number of subjects on NIV

Bach (45), 2003, USA	Quantitative: observational (case series)	n/a	3 (2/1)	*n* = 3	4–11 months	NIV (*n* = 3)		• Number of subjects on NIV • Benefit of NIV (growth parameters)

Bach (46), 2007, USA	Quantitative: observational (cohort)	13	92 (n/a)	*n* = 92^d^	Therapy group: none: 6.6 ± 4.1 months bi-level: 10.6 ± 5.7 months IMV: 14.8 ± 15.2 months	None (*n* = 18) BPAP (*n* = 47) IMV (*n* = 27)	• Hospitalizations • Survival/mortality	• Number of subjects on NIV

Barnerias (47), 2014, France	Quantitative: observational (cross-sectional)	20 years	222 (n/a)	*n* = 8	Overall: 3 months (0.5–8 months)	NIV (*n* = 8)		• Number of subjects on NIV*

Birnkrant (48), 1998, USA	Quantitative: observational (case series)	2 years	4 (3/1)	*n* = 3	4–9 months	BPAP (*n* = 4)	• Survival/mortality	• Number of subjects on NIV • Benefit of NIV (extubation)

Chatwin (49), 2011, UK	Quantitative: observational (cohort)	19 years	13 (8/5)	*n* = 13	4–24 months	BPAP (*n* = 13)	• Survival/mortality	• Number of subjects on NIV • Benefit of NIV (growth parameters)

Ednick (50), 2008, USA	Quantitative: observational (cohort)	3.5 years	7 (1/6)	*n* = 7	8.3 ± 3.7 months	BPAP (*n* = 7)		• Number of subjects on NIV • Benefit of NIV (extubation)

Gregoretti (51), 2013, Italy	Quantitative: observational (case series)	18 years	194 (103/91)	*n* = 31	NIV group: 12.6 ± 14.4 months (0–42 months) IMV group: 6.9 ± 4.3 months	None (*n* = 121) NIV (*n* = 31) IMV (*n* = 42)	• Hospitalizations • Survival/mortality	• Number of subjects on NIV

Ioos (52), 2004, France	Quantitative: observational (cohort)	n/a	180 (n/a)	*n* = 33	19 ± 17 months	n/a		• Number of subjects on NIV*

Lemoine (53), 2012, USA	Quantitative: observational (cohort)	7 years	49 (31/18)	*n* = 49	Groups: NIV: 136 days (34–196 days) Supportive care: 69 days (38–145 days)	None (*n* = 23) BPAP (*n* = 26)	• Hospitalizations • Survival/mortality	• Number of subjects on NIV

Ottonello (54), 2011, Italy	Quantitative: observational (cohort)	4 years	16 (n/a)	*n* = 14^e^	Overall: <3 years Infants: 10.4 ± 6.2 months	NIV (*n* = 16)	• Hospitalizations • Survival/mortality	• Number of subjects on NIV • Benefit of NIV

Petrone (55), 2007, Italy	Quantitative: control before–after	n/a	9 (7/2)	*n* = 9^d^	7 months (2–33 months)	BPAP (*n* = 9)	• Changes in respiratory parameters	• Number of subjects on NIV

Vasconcelos (56), 2005, Portugal	Quantitative: observational (cohort)	11 years	22 (16/6)	*n* = 7^d^	Overall: 5.5 years (6 months to 26 years) SMA type 1 group: 13 months (3 months to 3 years)	None (*n* = 5) BPAP (*n* = 17)	• Hospitalizations • Survival/mortality	• Number of subjects on NIV • Benefit of NIV (growth parameters)

**Articles on neuromuscular disease: achondroplasia**								

Afsharpaiman (57), 2011, Iran, Australia	Quantitative: observational (cohort)	15 years	46 (22/24)	*n* = 7	Overall: 3.9 years Infants: <2 years (*n* = 7)	CPAP (*n* = 9) AT (*n* = 13)		• Number of subjects on NIV*

**Articles on neuromuscular disease: multiple (spinal muscular atrophy type 1 and congenital myopathy)**								

Han (58), 2015, Korea	Quantitative: observational (cohort)	13.4 years	57 (n/a)	n/a	Overall: 7.7 months (2–158 months) Infants with SMA type 1: 6.6 months (2–26) CM: 7.8 months (3–121)	NIV (*n* = 8) IMV (*n* = 46)	• Survival/mortality	• Number of subjects on NIV • NIV success/failure

**Articles on neuromuscular disease: myotonic dystrophy**								

Wood (59), 2017, UK, Germany	Quantitative: observational (cross-sectional)	4 years	610 (272/338)	*n* = 2	41.1 years (8 months to 78 years)	NIV (*n* = 35)		• Number of subjects on NIV*

**Articles on central nervous system disease: congenital hypoventilation syndrome**								

Garcia Teresa (60), 2017, Spain	Quantitative: observational (cross-sectional)	3.75 years	38 (17/21)	*n* = 8^d^	11.35 (5 months to 28.6 years)	NIV (*n* = 8)	• Hospitalizations • Survival/mortality	• Number of subjects on NIV • NIV failure/success

Hartmann (61), 1994, UK	Quantitative: observational (case series)	n/a	9 (3/6)	*n* = 6	22 days to 52 months	VNEP (*n* = 9)^f^ CPAP (*n* = 3)^g^	• Discontinuation of NIV	• Number of subjects on NIV • Benefit of NIV (growth parameters) • NIV success/failure Quality of life

Khayat (62), 2017, Canada, USA	Quanitative: observational (control before–after)	2.7 years	8 (4/4)	*n* = 2	Overall: 10.0 years (8.4–11.6 years) Infants: 1.1 years	BPAP (*n* = 8)^h^		• Number of subjects on NIV • NIV modality

Noyes (63), 1999, UK, Germany	Qualitative: content analysis	n/a	7 (3/4)	*n* = 5	66 days to 59 months	VNEP (*n* = 5) CPAP (*n* = 1)^g^ IMV (*n* = 2)	• Discontinuation of NIV	• Number of subjects on NIV • Benefit of NIV (growth parameters) • Quality of life

Ramesh (64), 2008, UK	Quantitative: observational (cross-sectional)	n/a	15 (5/10)	*n* = 7	Early start: 8 weeks (5–26 weeks) Late start: 8 years (1.5–11 years)	NIV (*n* = 15)		• Number of subjects on NIV • Benefit of NIV (extubation) • Mask complications

Tibballs (65), 2003, Australia	Quantitative: observational (case series)	n/a	4 (2/2)	*n* = 2	6 weeks to 9 years	BPAP (*n* = 4)	• Changes in respiratory parameters	• Number of subjects on NIV • Benefit of NIV (extubation) • Mask complications

**Articles on cardio-respiratory disease: congenital heart disease**								

Bunn (66), 2004, UK	Quantitative: observational (case series)	n/a	4 (0/4)	*n* = 3	5–34 months	NIV (*n* = 4)	• Changes in respiratory parameters • Discontinuation of NIV	• Number of subjects on NIV

**Articles on multiple underlying disease conditions**								

Adeleye (67), 2016, Canada	Quantitative: observational (cohort)	5 years	92 (54/38)	*n* = 49	208.5 ± 101.2 days	NIV (*n* = 49)		• Number of subjects on NIV • Adherence to NIV

Amaddeo (3), 2016, France	Quantitative: observational (cohort)	1 year	76 (39/37)	n/a	Overall for patient groups: acute: 0.3 year (0.1–13.5) Sub-acute: 0.6 year (0.2–18.2) Chronic: 1.6 years (0.1–19.5)	CPAP (*n* = 64) BPAP (*n* = 12)		• Number of subjects on NIV • Predictors of NIV requirement

Bertrand (68), 2006, Chile	Quantitative: observational (cohort)	10.5 years	35 (18/17)	*n* = 9^d^	12 months (5 months to 14 years)	CPAP (*n* = 1) BPAP (*n* = 8) IMV (*n* = 26)	• Hospitalizations • Discontinuation of NIV • Survival/Mortality	• Number of subjects on NIV

Chatwin (7), 2015, UK	Quantitative: observational (cohort)	18 years	449 (281/168)	*n* = 59^c^	Overall: 10 years (3–15 years) Infants: <1 year (*n* = 59)	CPAP (*n* = 57) BPAP (*n* = 392)		• Number of subjects on NIV*

Fauroux (69), 2005, France	Quantitative: observational (cross-sectional)	0.5 year	40 (22/18)	*n* = 16	Overall: 10.0 years (0.6–18 years) Infant: 1.8 years (0.2–15.3 years)^i^	NIV (*n* = 40)		• Number of subjects on NIV • Adherence to NIV • Mask complications

Kherani (70), 2016, Canada	Quantitative: observational (cohort)	23 years	51 (30/21)	*n* = 25	NIPPV: 0.6 year (0.4–0.7 year) IMV: 0.4 year (0.1–0.7 year)	NIV (*n* = 25) IMV (*n* = 26)	• Changes in respiratory parameters • Discontinuation of NIV • Survival/mortality	• Number of subjects on NIV

Koontz (71), 2003, USA	Quantitative: observational (cohort)	n/a	20 (n/a)	*n* = 6	1–2 years	BPAP (*n* = 6)		• Number of subjects on NIV • Adherence to NIV

Machaalani (72), 2016, Australia	Quantitative: observational (cohort)	2 years	99 (63/36)	*n* = 22	n/a	CPAP (*n* = 55) BPAP (*n* = 44)		• Number of subjects on NIV*

Markstrom (9), 2008, Sweden	Quantitative: observational (cohort)	7 years	18 (11/7)	*n* = 18	4 months (1–12 months)	BPAP (*n* = 18)	• Changes in respiratory parameters • Discontinuation of NIV	• Number of subjects on NIV

Nathan (73), 2017, Malaysia	Quantitative: observational (cohort)	13 years	70 (40/30)	*n* = 51	Overall: 12 months CPAP: 6 months (3–12 months) BPAP: 12 months (5–33 months) IMV: 30 (12–57 months)	CPAP (*n* = 30) BPAP (*n* = 30) IMV (*n* = 10)	• Discontinuation of NIV • Hospitalizations • Survival/mortality	• Number of subjects on NIV • Predictors of NIV • NIV modality

Ramirez (74), 2012, France	Quantitative: observational (case series)	18 months	97 (n/a)	*n* = 18	Infants: <2 years (*n* = 18)	CPAP and BPAP (n/a)		• Number of subjects on NIV*

Zhou (75), 2012, China	Quantitative: observational (cohort)	2 years	14 (12/2)	*n* = 6^c^	Overall: 50 days to 12 years Infants: <1 year (*n* = 6)	CPAP (*n* = 1) BPAP (*n* = 13)		• Number of subjects on NIV*

*Studies have been classified according to the primary disease category and disease condition reported. Studies with multiple disease categories have been included at the end of the table*.

*AT, adenotonsillectomy; CPAP, continuous positive airway pressure; BPAP, bi-level positive airway pressure; IMV, invasive mechanical ventilation; n/a, data not available/reported; MDO, mandibular distraction osteogenesis; NIV, non-invasive ventilation; NPA, nasopharyngeal airway; SMA, spinal muscular atrophy; VNEP, negative extra-thoracic pressure ventilation*.

*Articles reporting only on the number of subjects using NIV were excluded from synthesis.

*^a^Four patients did not tolerate CPAP*.

*^b^Full list of non-surgical and surgical interventions are in the full text of article*.

*^c^Number of patients less than 1 year of age*.

*^d^Determined by the mean/median age of the population during NIV initiation*.

*^e^Determined by age at first respiratory decompensation*.

*^f^VNEP failed in two patients*.

*^g^CPAP used in conjunction with VNEP*.

*^h^Compared intelligent volume-assured pressured support BPAP to traditional BPAP*.

*^i^Only includes infants in the obstructive sleep apnea group*.

The majority of studies were retrospective (41/60, 68%), quantitative (59/60, 98%), and single-center studies (54/60, 90%). The most common study design was observational, which included cohort studies (31/60, 52%), case series (13/60, 25%), and cross-sectional studies (8/60, 13%). Forty-eight percent of studies were exclusively on the infant population. Based on primary underlying disease categories, the studies were distributed across airway disorders (24/60, 40%), NMD (17/60, 28%), CNS (6/60, 10%), cardio-respiratory diseases (1/60, 2%), and multiple disease categories (12/60, 20%; Table 2). Thirteen studies did not report NIV outcomes, only the number of infants using NIV, and were excluded from further analysis (7, 25, 26, 32, 33, 37, 47, 52, 57, 59, 72, 74, 75).

#### Obstructive Sleep Apnea

Obstructive sleep apnea (OSA) was the most common airway disorder studied in the infant population, with 12 studies (12/60, 20%) reporting on this condition (Table 2). Of these, 10 studies reported on infant NIV outcomes and were synthesized in the review (10, 20–24, 27–30). These studies included infants with multiple underlying conditions, the most common being a history of acute life-threatening events (ALTE), family history of sudden infant death syndrome (SIDS), and craniofacial malformations. Eight studies (8/10, 80%) reported on changes in respiratory parameters, with seven of these studies (7/10, 70%) showing improvements in central, obstructive, and/or mixed apneas from a diagnostic to titration polysomnography (Table 3) (10, 20, 22, 23, 27–29) Only one study (1/10, 10%) included diagnostic polysomnography results after long-term NIV use (weighted mean of 12 months), which showed an overall decrease in respiratory events, normalization of respiratory gases, and increased arousals during REM sleep (29). Five studies (5/10, 50%) reported discontinuation of NIV in infants because of improvements in respiratory parameters, with discontinuation rates ranging from 14 to 100% (weighted mean 70 ± 26%) (20, 21, 27, 29, 30). No studies reported on hospitalization outcomes (Table 4). One study (1/10, 10%) of five infants using NIV reported mortality outcomes, with all infants alive at the time of study publication (27).

**Table 3:** Studies on infants using long-term NIV reporting change in respiratory parameters and discontinuation outcomes.

First author, year, country	Study design	Primary diagnosis	Infants using NIV	Age mean ± SD or med (range)	NIV type	Total apneas (mean ± SD events/hour)		Obstructive apneas (mean ± SD events/hour)		Central apneas (mean ± SD events/hour)		Infants who discontinued (%)

						Pre-NIV	Post-NIV	Pre-NIV	Post-NIV	Pre-NIV	Post-NIV	
Harrington (22), 2003, Australia, Finland	P, Obs: case–control	OSA	*n* = 6	13 ± 4 weeks	CPAP			17 ± 6	1 ± 1*			

Downey (20), 2000, USA	R, Obs: cohort	OSA	*n* = 18	<2 years	CPAP	12.8 ± 20.0	4.5 ± 13.4^†^	4.7 ± 13.4	2.0 ± 7.3^†^			90

McNamara (27), 1995, Australia	P, control before–after	OSA	*n* = 5	8–12 weeks	CPAP	^a^65.6 ± 14.6^b^106.1 ± 13.9	10.5 ± 14.6**26.6 ± 13.9**	^a^29.3 ± 9.4^b^80.8 ± 16.8	^a^0.3 ± 9.4**^b^2.0 ± 16.8**	36.5 ± 6.625.6 ± 4.5	10.3 ± 6.6**24.6 ± 4.5	100

McNamara (28), 1999, Australia	P, Obs: cohort	OSA	*n* = 24	1–51 weeks	CPAP	44.4 ± 9.368.6 ± 8.9	9.5 ± 1.2*22.7 ± 2.3*	14.6 ± 3.943.6 ± 8.3	0.1 ± 0.1*0.4 ± 0.1*	29.8 ± 7.625.0 ± 4.3	9.4 ± 1.2*22.3 ± 2.2	72

McNamara (29), 1999, Australia	P, Obs: case–control	OSA	*n* = 8	10.8 ± 1.3 weeks	CPAP			22.2 ± 8.854.8 ± 16.3	10.6 ± 2.6*25.7 ± 7.2*	36.1 ± 8.632.9 ± 8.1	26.3 ± 7.438.2 ± 8.2	

Leonardis (23), 2013, USA	R, Obs: cohort	OSA	*n* = 18	16.0 mo	CPAP BPAP	% decrease in AHI: 67.2*						

Robison (10), 2013, USA	R, Obs: cohort	OSA	*n* = 18	15.6 months (3–29 months)	CPAP BPAP	% decrease in AHI: 84.1*						

Guilleminault (21), 1995, USA	P, Obs: case–control	OSA	*n* = 72	24 ± 9 weeks (4–43 months)	CPAP							14

Rosen (30), 2010, United States	R, Obs: cohort	OSA	*n* = 6	<2 years	CPAP							50

**First author, year, country**	**Study design**	**Primary Diagnosis**	**Infants using NIV**	**Age, mean ± SD or med (range)**	**NIV type**	**Change in respiratory parameters**						**Infants who discontinued (%)**
						**Variables**				**Pre-NIV, mean ± SD**	**Post-NIV, mean ± SD**	

Leboulanger (36), 2010, France	P; Obs: case series	PRS	*n* = 7	2 months (1–10 months)	CPAP (*n* = 5) BPAP (*n* = 2)	RR (breaths/minute) *T*_I_/*T*_TOT_ (%) *P*_es_ swing (cm H_2_O) *P*_di_ swing (cm H_2_O) Total sleep time with S_p_O_2_ < 90% (%) Total sleep time with P_a_CO_2_ > 50 mm Hg (%)				55 ± 9 59 ± 9 29 ± 13 31 ± 12 14 ± 10 88 ± 12	37 ± 7 40 ± 7* 9 ± 4* 12 ± 5* 1 ± 2* 0 ± 0^†^	71

Amaddeo (31), 2016, France	R; Obs: cohort	PRS	*n* = 9	0–2 months	CPAP	Apnea–hypopnea index (events/hour) Oxygen desaturation index (events/hour) Minimum S_p_O_2_ (%) % time S_p_O_2_ < 90% Maximum P_a_CO_2_ (%)				19–42 18–137 78–90 0–16 41–55	Normal PG and/or gas exchange (reported narratively)	66

**First author, year, country**	**Study design**	**Primary diagnosis**	**Infants using NIV**	**Age, mean ± SD or med (range)**	**NIV type**	**Variables**		**Supportive care**		**CPAP**	**BPAP**	**Infants who discontinued (%)**

Essouri (38), 2005, France	P; Obs: case–control	LTM	*n* = 10	9.5 months (3–18 months)	CPAP (*n* = 10)BPAP (*n* = 10)	RR (breaths/minute) *T*_I_/*T*_TOT_ (%) *P*_es_ swing (cm H_2_O) *P*_di_ swing (cm H_2_O) PTP_es_/minute (cm H_2_O/second/minute) PTP_di_/minute (cm H_2_O/second/minute)		45 (24–84) 63 (35–86) 28 (13–76) 30 (16–75) 695 (364–1417) 845 (159–1183)		29 (18–60) 41 (34–60)** 10 (7–28)** 12 (8–32)** 143 (98–469)** 195 (115–434)**	25 (14–50)^**c^ 48 (28–55)** 13 (6–33)** 14 (7–33)** 211 (73–588)** 248 (45–784)**	

Fauroux (39), 2001, France, UK	P; Obs: case–control	LTM	*n* = 5	8–19 months	CPAP	S_p_O_2_ (%) S_p_O_2_ nadir (%) % sleep with S_p_O_2_ < 90%		91.7 ± 2.3 74.7 ± 7.5 29.5 ± 19.6		96.2 ± 2.0* 88.0 ± 2.5* 0.5 ± 0.8*		60

Zwacka (41), 1997, Germany	R: Obs: cohort	LTM	*n* = 7	3 weeks to 3 months	CPAP	HR (beats/minute) RR (breaths/minute) S_a_O_2_ in REM sleep (%) S_a_O_2_ in NREM sleep (%)		135–160 34–42 60–95 85–98		110–130 22–28 88–100 92–100		

Shatz (40), 2004, Israel	R; Obs: cohort	LTM	*n* = 14	6.5 ± 3.5 months (1–18 months)	CPAP (*n* = 5) BPAP (*n* = 9)							100

**First author, year, country**	**Study design**	**Primary diagnosis**	**Infants using NIV**	**Age, mean ± SD or med (range)**	**NIV type**	**Change in respiratory parameters**						**Infants who discontinued (%)**

Tibballs (65), 2003, Australia	R; Obs: case series	CHS	*n* = 2	6 weeks and 9 months	BPAP (*n* = 2) VNEP (*n* = 2)	Decrease in P_a_CO_2_ to 40–50 mm Hg in one infant						

Hartmann (61), 1994, U	P; Obs: case series	CHS	*n* = 6	22 days to 5 months	VNEP (*n* = 6) CPAP (*n* = 2)	Improvements in hypoventilation in three patients (reported narratively)						33

Noyes (63), 1999, UK	P; Obs: cross-sectional	CHS	*n* = 5	66 days to 59 months	VNEP (*n* = 5) CPAP (*n* = 1)							33

Ramesh (64), 2008, UK	P; Obs: cross-section	CHS	*n* = 6	8 weeks (5–26 weeks)								0

*AHI, apnea–hypopnea index (events/hour); BPAP, bi-level positive airway pressure; CHS, congenital hypoventilation syndrome; CPAP, continuous positive airway pressure; HR, heart rate; LTM, laryngo-tracheomalacia; NIV, non-invasive ventilation; Obs, observational study; OSA, obstructive sleep apnea; P, prospective; P_a_CO_2_, partial pressure of carbon dioxide; *P*_di_, diaphragmatic pressure; *P*_es_, esophageal pressure; PG, polygraphy; PRS, Pierre Robin sequence; R, retrospective; RR, respiratory rate; S_a_O_2_, oxygen saturation; SpO_2_, pulse oximetry; *T*_I_/*T*_TOT_, inspiratory time/total respiratory cycle time; VNEP, negative extra-thoracic pressure ventilation*.

*p < 0.05.

**p < 0.01.

*^†^p < 0.001*.

*^a^Apneas seen in non-rapid eye movement (NREM) sleep*.

*^b^Apneas seen in rapid eye movement (REM) sleep*.

**Table 4:** Studies on infants using long-term NIV reporting hospitalization and mortality outcomes.

First author, year, country	Study design	Primary diagnosis	Infants using NIV	Age, mean ± SD or med (range)	NIV type	Hospitalization (per infant/year unless otherwise stated)			Mortality (% of total infants unless otherwise stated)		
						Supportive care	NIV	IMV	Supportive care	NIV	IMV
Bach (46), 2007, USA	R; Obs: cohort	SMA1	*n* = 47	10.6 ± 5.7 months	BPAP		1.58	0.37^†^	100	17	19

Bach (44), 2002, USA	R; Obs: case series	SMA1	*n* = 33	11.2 ± 5.7 months	BPAP		1.53	0.58*	100	6	6

Gregoretti (51), 2013, Italy	R; Obs: case series	SMA1	*n* = 31	12.6 ± 14.4 months	BPAP		0.023	0.006	93	45	17

Ottonello (54), 2011, Italy	R; Obs: cohort	SMA1	*n* = 16	10.4 ± 6.2 months	BPAP		0.15			13	

Bach (43), 2000, USA	R; Obs: case series	SMA1	*n* = 8	3–28 months	BPAP					13	

Birnkrant (48), 1998, USA	R; Obs: case series	SMA1	*n* = 3	4–9 months	BPAP					100	

Chatwin (49), 2011, UK	R; Obs: cohort	SMA1	*n* = 13	11 months (4–24 months)	BPAP					38	

Vasconcelos (56), 2005, Portugal	R; Obs: cohort	SMA1	*n* = 7	13 months (3 months to 3 years)	BPAP					71	

Lemoine (53), 2012, USA	R; Obs: cohort	SMA1	*n* = 26	136 days (54–196)	BPAP	46%	83%		NIV group had a significantly longer survival than supportive care group (*p* = 0.047, reported narratively)		

**First author, year, country**	**Study design**	**Primary diagnosis**	**Infants using NIV**	**Age, mean ± SD or med (range)**	**NIV type**	**Length of hospital stay [mean ± SD or med (range)]**			**Mortality (% of total infants)**		

						**No ventilation**	**NIV**	**IMV**			

Leboulanger (36), 2010, France	P; Obs: case Series	PRS	*n* = 7	2 months (1–10 months)	CPAP (*n* = 5) BPAP (*n* = 2)						

Amaddeo (31), 2016, France	R; Obs: cohort	PRS	*n* = 9	0–2 months	CPAP		1 month (20–40 days)	2 months (6 weeks to 4 months)			

Kam (35), 2015, Canada	R; Obs: cohort	PRS	*n* = 20	23 months (5 days to 8 years)	CPAP	28 ± 24 days	66 ± 46 days	138 ± 76 days^†^	NR		

**First author, year, country**	**Study design**	**Primary diagnosis**	**Infants using NIV**	**Age, mean ± SD or med (range)**	**NIV type**	**Hospitalization (per infant/year or % of total)**			**Mortality (% of total infants)**		

McNamara (27), 1995, Australia	P, Obs: cohort	OSA	*n* = 5	8–12 weeks	CPAP	–			0%		

**First author, year, country**	**Study design**	**Primary diagnosis**	**Infants using NIV**	**Age, mean ± SD or med (range)**	**NIV type**	**Length of hospital stay [mean ± SD or med (range)]**			**Mortality**		
						**No ventilation**	**NIV**	**IMV**			

Garcia Teresa (60), 2017, Spain	P, Obs: cross-sectional	CHS	n/a	11.35 (5 months to 28.6 years)	NIV		91 ± 51 days	319 ± 336 days**	*n* = 2 infants		

*BPAP, bi-level positive airway pressure; IMV, invasive mechanical ventilation; NIV, non-invasive ventilation; Obs, observational study; OSA, obstructive sleep apnea; P, prospective; PRS, Pierre Robin sequence; R, retrospective; SMA1, spinal muscular atrophy type 1*.

*p < 0.05.

**p < 0.01.

*^†^p < 0.001*.

#### Pierre Robin Sequence

Seven studies (7/60, 12%) reported on infants with Pierre Robin sequence (PRS) using long-term NIV (Table 2). Four studies (4/7, 57%) reported on primary or secondary outcomes and were synthesized for this review (31, 34–36). A cohort study reported normalization of polygraphy parameters and gas exchange post-NIV initiation (Table 3) (31). A case series reported a decrease in respiratory rates, statistically significant improvements in respiratory effort, and normalization of respiratory gases after administration of NIV therapy in infants with PRS (36). Two studies on 16 infants with PRS reported discontinuation from NIV in 11 (69%) infants because of improvements in respiratory parameters (31, 36). Two studies comparing infants on NIV and invasive mechanical ventilation showed that the length of hospitalization were shorter for infants on NIV than for those receiving invasive mechanical ventilation via a tracheostomy (Table 4) (31, 35). No studies addressed survival outcomes in infants with PRS using long-term NIV. Adherence of infants to NIV was reported as excellent, showing more than 8 hours of NIV use per day in two studies (31, 36), with only a 1–2 week period required to adjust to the mask ventilation (31, 35). An additional cohort study demonstrated that infants with PRS using NIV were 10.43 times more likely to progress to a surgical airway compared to infants who required less advanced respiratory supports such as prone positioning and a nasopharyngeal airway (34).

#### Laryngo-Tracheomalacia

All four studies (4/60, 7%) on infants with laryngo-tracheomalacia (LTM) using long-term NIV reported on primary or secondary outcomes and were synthesized in the review (Table 2) (38–41). Three studies (3/4, 75%) reported on changes in respiratory parameters (Table 3) (38, 39, 41). A case–control study of 10 infants with LTM showed improvements in respiratory frequency and respiratory effort in infants using CPAP or BPAP compared to spontaneous breathing (38). Normalization of arterial oxygen saturations after NIV use was seen in two studies (39, 41). NIV discontinuation was reported in two studies, with a combined discontinuation due to improvement rate of 81% (13/16 infants) (39, 40). No studies examined hospitalization or mortality outcomes. Improvement in chest wall deformity after NIV use in three patients and normalization of weight in four patients was reported in one case–control study (39). The same study also reported an average NIV use per day of 10.2 hours/day in seven infants (50).

#### Spinal Muscular Atrophy Type 1

There were 14 studies (14/60, 23%) of infants with spinal muscular atrophy type 1 (SMA1) using long-term NIV (Table 2). Twelve of these studies reported on primary or secondary outcomes and were synthesized (43–46, 48–51, 53–56). Only one study (1/12, 8%) reported on changes in respiratory parameters and showed improvements in respiratory effort and normalization of respiratory gases in SMA1 patients using NIV therapy (Table 3) (55). Six studies (6/12, 50%) reported on hospitalization outcomes (Table 4) (43, 44, 46, 51, 53, 54). Of these, two studies reported that hospitalizations per patient per year were significantly higher in infants on NIV than infants with a tracheostomy until after three years of age (44, 46). Nine studies (9/12, 75%) reported on mortality outcomes (43, 44, 46, 48, 49, 51, 53, 54, 56); four of these studies compared infants on supportive care with those using NIV, showing prolonged survival in the NIV group (44, 46, 51, 53). Three studies (3/12, 25%) reported improvements in growth parameters, seen by resolution of chest wall deformity (pectus excavatum) after the initiation of NIV therapy (43, 45, 49). An additional three studies showed that NIV helped facilitate extubation in infants with SMA1 (43, 48, 50).

#### Central Hypoventilation Syndrome

There were six studies (6/60, 10%) on NIV use for infants with central hypoventilation syndrome (CHS) that reported primary or secondary outcomes, and all six were summarized (Table 2) (60–65). The diagnosis of CHS was confirmed clinically in two studies (61, 65), via PHOX2B gene mutation analysis in three studies (60, 62, 64), and unreported in one study (63). NIV was used in conjunction with negative extra-thoracic pressure ventilation (VNEP) therapy in two studies: in one study, it was used as the primary therapy (65) and, in the second study, CPAP was used to relieve upper airway obstruction not resolved with VNEP (61). Improvements in respiratory parameters were reported in two studies: one showed the normalization of the partial pressure of carbon dioxide and resolution of pulmonary hypertension following the use of NIV (65) and the other study showed improvements in hypoventilation for 50% (3/6) of infants (Table 3) (61). One study with six infants reported NIV discontinuation in two infants (33%) because of improvements in respiratory parameters; the remaining four infants were using NIV only during sleep (61). One cohort study reported mortality outcomes and a higher hospitalization time for infants using invasive mechanical ventilation compared to NIV (Table 4) (60). Two studies showed parent-reported improvements in growth and development after NIV initiation using the results of a parent questionnaire (61, 63). An additional two studies reported pressure-related effects of mask use, which were predominantly skin breakdown and mid-face hypoplasia (64, 65). One cross-sectional study showed that it took less than a week for five of the six infants to adjust to NIV (61). A control before-after study of infants using two BPAP ventilators showed comparable sleep and respiratory parameters with both ventilators, with the exception of a greater decrease in the maximum transcutaneous carbon dioxide with the intelligent volume-assured pressured support compared to a traditional BPAP ventilator (62).

#### Synthesized Findings

**Figure 2:**
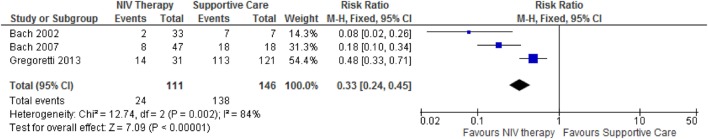
A meta-analysis on the effect of non-invasive ventilation (NIV) on the relative risk of mortality in infants with spinal muscular atrophy. The meta-analysis shows that the relative risk of mortality is significantly lower in infants using NIV compared to infants on supportive care. This decrease may be attributed to prolonged survival in infants using long-term NIV compared to supportive care.

After examining studies for all disease categories and respective outcomes, only three studies on infants with SMA1 reporting mortality outcomes were eligible for meta-analysis (44, 46, 51). The results of meta-analysis showed that there was a statistically significant decrease in the relative risk of mortality in the NIV group compared to the supportive care group (Figure 2).

### Risk of Bias and Quality Assessment of Outcomes

Risk of bias ranged from moderate to severe in all studies synthesized in this review (Table 5). Study design was the main contributor to the low quality assessment of the studies. Almost all the included studies had an observational study design, which contributed to confounding bias in participant selection and selected reporting of results. Grading of the quality of the evidence for outcomes such as changes in respiratory parameters, discontinuation of NIV, hospitalizations, and mortality showed that the quality of evidence ranged from low to very low for all studies (Table 6).

**Table 5:** Assessment of risk of bias in studies synthesized in the systematic review on long-term non-invasive ventilation in infants using the Risk of Bias in Non-randomized Studies of Interventions (ROBINS-I) tool (18).

First author, year	Confounding	Selection	Measurement of intervention	Missing data	Measurement of outcomes	Selection of reported results	Overall risk of bias (RoB) assessment^a^
**Obstructive sleep apnea**							

Downey (20), 2000	Moderate	Moderate	Serious	Serious	Serious	Serious	Serious
Guilleminault (21), 1995	Serious	Serious	Serious	Serious	Serious	Moderate	Serious
Harrington (22), 2003	Moderate	Moderate	Serious	Moderate	Moderate	Moderate	Serious
Leonardis (23), 2013	Moderate	Serious	Moderate	Serious	Serious	Moderate	Serious
Liu (24), 2012	Serious	Serious	Moderate	Moderate	Moderate	Serious	Serious
McNamara (27), 1995	Moderate	Moderate	Moderate	Serious	Moderate	Moderate	Serious
McNamara (28), 1999a	Moderate	Moderate	Moderate	Moderate	Moderate	Moderate	Moderate
McNamara (29), 1999b	Moderate	Moderate	Moderate	Moderate	Moderate	Moderate	Moderate
Robison (10), 2013	Moderate	Moderate	Serious	Serious	Serious	Moderate	Serious
Rosen (30), 2010	Moderate	Serious	Serious	Serious	Serious	Serious	Serious

**Pierre Robin sequence**							

Amaddeo (31), 2016	Serious	Serious	Serious	Moderate	Serious	Moderate	Serious
Kam (35), 2015	Moderate	Moderate	Serious	Serious	Moderate	Moderate	Serious
Leboulanger (36), 2010	Moderate	Moderate	Serious	Moderate	Moderate	Moderate	Serious
Goudy (34), 2017	Serious	Serious	Serious	Moderate	Serious	Moderate	Serious

**Laryngo-tracheomalacia**							

Essouri (38), 2005	Moderate	Moderate	Moderate	Moderate	Low	Low	Moderate
Fauroux (39), 2001	Moderate	Moderate	Moderate	Serious	Moderate	Moderate	Serious
Shatz (40), 2004	Moderate	Serious	Serious	Serious	Serious	Moderate	Serious
Zwacka (41), 1997	Serious	Serious	Serious	Serious	Serious	Serious	Serious

**Spinal muscular atrophy type 1**							

Bach (43), 2000	Serious	Serious	Serious	Serious	Serious	Serious	Serious
Bach (44), 2002	Serious	Serious	Serious	Serious	Serious	Serious	Serious
Bach (46), 2007	Serious	Serious	Serious	Serious	Low	Moderate	Serious
Birnkrant (48), 1998	Serious	Serious	Serious	Moderate	Serious	Serious	Serious
Chatwin (49), 2011	Serious	Serious	Serious	Moderate	Moderate	Serious	Serious
Gregoretti (51), 2013	Moderate	Moderate	Moderate	Moderate	Moderate	Moderate	Moderate
Lemoine (53), 2012	Moderate	Serious	Serious	Moderate	Moderate	Moderate	Serious
Ottonello (54), 2011	Moderate	Serious	Serious	Moderate	Moderate	Moderate	Serious
Vasconcelos (56), 2005	Serious	Serious	Serious	Serious	Serious	Serious	Serious

**Congenital hypoventilation syndrome**							

Hartmann (61), 1994	Serious	Serious	Serious	Serious	Serious	Serious	Serious
Noyes (63), 1999	Serious	Serious	Serious	Serious	Serious	Serious	Serious
Ramesh (64), 2008	Moderate	Serious	Serious	Moderate	Serious	Serious	Serious
Tibballs (65), 2003	Moderate	Serious	Serious	Moderate	Serious	Serious	Serious
García Teresa (60), 2017	Serious	Serious	Serious	Moderate	Serious	Serious	Serious
Khayat (62), 2017	Serious	Serious	Serious	Moderate	Serious	Moderate	Serious

*Low risk of bias—study is comparable to a well performed randomized trial within that domain. Moderate risk of bias—study is sound for a non-randomized study, but is not considered comparable to a well performed randomized trial within that domain. Serious risk of bias—study has some important problems within that domain. Critical risk of bias—the study is too problematic in this domain to provide any useful evidence on the effects of intervention. No information—no information on which to base a judgment about risk of bias within that domain*.

*^a^Criteria set out by the ROBINS-I tool*.

**Table 6:** Quality assessment of outcomes of infants using long-term non-invasive ventilation using the Grading of Recommendations Assessment, Development and Evaluation criteria (19).

Quality assessment							Number of patients		Effect		Quality	Importance
Number of studies	Study design	Risk of bias^a^	Inconsistency	Indirectness	Imprecision	Other considerations	Intervention	Control	Relative (95% CI)	Absolute (95% CI)		
**Obstructive sleep apnea**												

**Changes in respiratory parameters: respiratory gases pre-NIV to post-NIV**												
5 (20, 22, 27–29)	Observational studies	Serious	Not serious	Not serious	Not serious	None	53	53			⊕⊕○○ low	Important
3 (10, 23, 24)	Observational studies	Serious	Not serious	Not serious	Not serious	None	–	–			⊕○○○ very low	Important
**Discontinuation of NIV**												
5 (20, 21, 27, 28, 30)	Observational studies	Serious	Not serious	Not serious	Not serious	None	–	–			⊕⊕○○ low	Important

**Pierre Robin sequence**												

**Changes in respiratory parameters: respiratory gases pre-NIV to post-NIV**												
2 (31, 36)	Observational study	Serious	Not serious	Not serious	Not serious	None	–	–			⊕○○○ very low	Important
**Discontinuation of NIV**												
2 (31, 36)	Observational studies	Serious	Not serious	Not serious	Not serious	None	–	–			⊕○○○ very low	Important
**Length of hospitalization**												
2 (31, 35)	Observational studies	Serious	Not serious	Not serious	Not serious	None	–	–			⊕○○○ very low	Important
**Adherence**												
2 (31, 36)	Observational studies	Serious	Not serious	Not serious	Not serious	None	–	–			⊕○○○ very low	Important

**Laryngo-tracheomalacia**												

**Changes in respiratory parameters: respiratory gases: supportive care vs. NIV**												
3 (38, 39, 41)	Observational studies	Serious	Not serious	Not serious	Not serious	None	24	24			⊕⊕○○ low	Important
**Discontinuation of NIV**												
2 (39, 40)	Observational studies	Serious	Not serious	Not serious	Not serious	None	–	–			⊕○○○ very low	Important
**Benefit of NIV—improvement in growth parameter(s)**												
1 (39)	Observational study	Serious	Not serious	Not serious	Not serious	None	–	–			⊕○○○ very low	Important

**Benefit of NIV—improvement in underlying condition(s)**												
1 (40)	Observational study	Serious	Not serious	Not serious	Not serious	None	–	–			⊕○○○ very low	Important
**Adherence**												
1 (39)	Observational study	Serious	Not serious	Not serious	Not serious	None	–	–			⊕○○○ very low	Important

**Spinal muscular atrophy type 1**												

**Mortality: NIV vs. supportive care**												
3 (44, 46, 51)	Observational studies	Serious	Not serious	Not serious	Not serious	None	24/111 (21.6%)	138/146 (94.5%)	RR 0.37 (0.25–0.54) *z* = 5.16 *p* < 0.0001	595 fewer per 1000 (from 435 fewer to 709 fewer)	⊕⊕○○ low	Very important
6 (43, 48, 49, 53, 54, 56)	Observational studies	Serious	Not serious	Not serious	Not serious	None					⊕⊕○○ low	Very important
**Hospitalization: per patient/per year**												
3 (43, 46, 51)	Observational studies	Serious	Not serious	Not serious	Not serious	None	–	–			⊕⊕○○ low	Very important
3 (43, 53, 54)	Observational studies	Serious	Not serious	Not serious	Not serious	None					⊕○○○ very low	Important
**Benefit of NIV—improvement in growth parameter(s)**												
3 (44, 46, 54)	Observational studies	Serious	Not serious	Not serious	Not serious	None	–	–			⊕⊕○○ low	Important
**Benefit of NIV—NIV facilitated extubation**												
3 (43, 48, 50)	Observational study	Serious	Not serious	Not serious	Not serious	None	–	–			⊕○○○ very low	Important
**Changes in respiratory parameters: respiratory gases**												
1 (55)	Observational study	Moderate	Not serious	Not serious	Not serious	None	–	–			⊕⊕○○ low	Important

**Congenital hypoventilation syndrome**												

**Changes in respiratory parameters: changes in respiratory gases post-NIV initiation**												
2 (61, 65)	Observational study	Serious	Not serious	Not serious	Not serious	None	–	–			⊕⊕○○ very low	Important

**Discontinuation of NIV**												
2 (61, 64)	Observational studies	Serious	Not serious	Not serious	Not serious	None	–	–			⊕○○○ very low	Important
**Benefit of NIV—improvement in growth parameter(s)**												
2 (61, 63)	Observational studies	Serious	Not serious	Not serious	Not serious	None	–	–			⊕○○○ very low	Important
**Mask complication(s)**												
2 (64, 65)	Observational studies	Serious	Not serious	Not serious	Not serious	None	–	–			⊕○○○ very low	Important
**Adherence**												
1 (61)	Observational study	Serious	Not serious	Not serious	Not serious	None	–	–			⊕○○○ very low	Important

*CI, confidence interval; n/a, data not available; NIV, non-invasive ventilation; RR, risk ratio*.

## Discussion

### Summary of Main Findings

To our knowledge, this is the first systematic review on the use of long-term NIV in infants. We identified studies on a diverse range of airway conditions in which NIV therapy improved the results of polysomnographic and respiratory parameters. With data available for NMD and CNS disorders limited to SMA1 and CHS, extrapolation of NIV benefits to other NMD and CNS disorders in infants is challenging. Not all outcomes were studied in all disease categories; length of hospitalization was the focus in studies of PRS, while hospitalizations and mortality were the focus in studies of SMA, and respiratory events and NIV discontinuation in the remaining groups. The overall quality of evidence to support appropriate conclusions was low to very low for all studies included in this review.

There is a diverse range of airway disorders that may benefit from NIV therapy. Previous studies have identified many conditions that can predispose infants to upper airway obstruction, including craniofacial disorders, laryngeal disorders, and nasal obstruction (76). Similarly, in this review, we identified NIV use in a wide variety of diseases associated with compromised airway function, the most common being OSA, PRS, ALTE, infants at risk for SIDS, and LTM. The improvement in respiratory parameters reported in infants with airway disorders reflects an overall benefit from NIV therapy. In addition, the underlying airway conditions have potential for improvement, as seen with the infants discontinuing due to underlying improvements, so there may be less risk with NIV compared to invasive mechanical ventilation. Extrapolating these results to conditions with a similar pathophysiology, but for which there is no evidence for NIV use in the literature, may be reasonable given the diversity of disorders represented in the available evidence.

By contrast, extrapolation of outcomes for long-term NIV use in NMD and CNS disorders may be more challenging. The data relevant to long-term NIV use for NMD and CNS disorders are almost exclusively from two conditions: SMA1 and CHS. SMA1 is a progressively deteriorating disorder that is usually fatal during infancy. This contrasts with other NMD disorders presenting in infancy, such as congenital myopathy and congenital muscular dystrophy, which may have a better prognosis or steadier course (7, 58). The difference in prognoses of these conditions makes generalizing outcomes for NIV use in SMA1 to other NMD less appropriate. Similarly, CHS was the only CNS disorder for which data on long-term NIV use was available. NIV may be useful for other CNS disorders with accompanying respiratory compromise, such as congenital or acquired brain injury. Given the potentially unique physiology of CHS extrapolating the outcomes of NIV use for infants with CHS to other CNS conditions with different underlying respiratory pathophysiology may not be appropriate. Creation of national disease registries for infants and children using NIV will provide the opportunity to aggregate data on rare or minimally studied diseases and examine the use and outcomes of long-term NIV in these populations.

The outcomes that were reported in studies differed depending on the primary underlying disease category that was being examined. Studies of airway conditions predominantly reported on changes in respiratory parameters reported via polysomnography results and discontinuation of NIV. In addition, most studies reported short-term overnight polysomnography results; only one study had data on polysomnography results after long-term follow-up periods of NIV use in infants (29). Only one study on upper airway disorders reported on mortality outcomes (27) and none on hospitalization outcomes. Long-term outcomes, such as hospitalizations, intercurrent illness, growth and development, and quality of life warrant further study. Interestingly, studies on SMA1 predominantly reported on mortality and hospitalization outcomes, with only one study reporting on changes in respiratory parameters.

While the overall quality of the evidence available for the use of long-term NIV in infants is low to very low, there is a body of evidence that may help guide clinical practice. The reason for the low quality of the evidence included the study design and a high risk of bias due to the lack of blinding and randomization, and control for confounding variables. While these findings highlight the need for future studies of strong design and lower risk of bias, the available data still provide important information to inform treatment decisions for conditions where long-term NIV is being considered.

### Limitations of the Included Studies

We identified a number of research gaps present in the studies included within this review. There was only one study that compared the efficacy of CPAP and BPAP ventilation in a cohort of infants (38). Similarly, while some studies reported mask complications (9, 21), only one compared the efficacy and practicality of different infant NIV masks (74). Only single studies were identified on the use of long-term NIV for infants with breath holding (42) and cardiac disease (66). Additionally, there were no studies on the clinical supports necessary for infants to be placed on NIV. It is important to know whether infants receive consultation and support from physicians, registered nurses, home care support, or a combination thereof, to determine whether a multidisciplinary NIV care plan is necessary for this population. The lack of comparison groups and/or homogeneity of outcomes reported precluded meta-analysis for most topics.

Additional issues relevant to long-term NIV use in infants that are not addressed in the current literature include: limitations in availability of masks and headgear; limitations in the availability of BPAP machines that are sufficiently sensitive to detect flow rates; the impact of NIV use on craniofacial growth and the impact of craniofacial growth on NIV use; co-morbidities in infants using NIV; the impact of NIV on somatic growth and psychomotor development; and, most importantly, the impact of NIV use on quality of life for both infants and caregivers.

### Limitations of the Review

Our review relied on the search methods and primary-level screening decisions of a scoping review on NIV in children with subsequent development of the research questions on NIV in infants. The methods to identify studies for the scoping review, however, were sufficiently inclusive to capture all relevant evidence on NIV in infants. We defined NIV for the scoping review on long-term NIV as breathing support outside the airway via an interface, consistent with the MeSH terminology for NIV and, therefore, included CPAP as well as BPAP. Some investigators, however, do not consider CPAP as a mode of NIV because it requires spontaneous breathing from the patient (1, 77). To address this concern, we reported the different ventilation types used by infants in the tables included in this review. Finally, we defined infants as ages 0–2 years based on the Public Health Agency of Canada definition (14). Some investigators may not agree with this definition, as the Centre for Disease Control defines infants as less than one year of age (78). Regardless of the definition used, it is still unclear whether there are differences in the outcomes of pediatric NIV with respect to age. Future work should consider whether infants represent a distinct group within children using long-term NIV.

## Conclusion

This systematic review examines the use and outcomes of long-term NIV in infants across a range of respiratory and sleep disorders. Improvements in respiratory parameters and discontinuation from NIV due to improvement in underlying conditions have been shown for a broad range of upper airway disorders, such as OSA, PRS, and LTM, in infants. Long-term NIV use in infants with SMA1 decreased hospitalizations and prolonged survival compared to infants on supportive care. Infants with CHS may also show improvements in respiratory parameters after using NIV and potentially avoid tracheostomy. NIV appears to be a feasible method of providing long-term respiratory support for infants with a wide range of underlying conditions; however, several methodological weaknesses limit any strong categorical conclusions. The findings of this systematic review are relevant to a broad range of stakeholders and can be used to help guide clinicians on the use of long-term NIV in infants.

## Author Contributions

PB conceptualized and designed the review, assessed articles for inclusion, extracted and analyzed data, interpreted the data, drafted the initial manuscript, and completed all subsequent revisions until submission. MC conceptualized and designed the review, assessed articles for inclusion, verified data extraction, and critically reviewed the manuscript. RF developed the search strategy, carried out the literature searches, and critically reviewed the manuscript. MA and BA assessed articles for inclusion, and critically reviewed the manuscript. AK provided guidance on study design and critically reviewed the manuscript. CF provided guidance on study design and review methodology and critically reviewed the manuscript. JM conceptualized and designed the review, assessed articles for inclusion, verified data extraction, interpreted the data, and critically reviewed the manuscript. All authors reviewed the manuscript and approved the final manuscript for submission.

## Conflict of Interest Statement

The authors declare that the research was conducted in the absence of any commercial or financial relationships that could be construed as a potential conflict of interest. The reviewer MP and handling editor declared their shared affiliation.

## Abbreviations

ALTE, acute life-threatening events; BPAP, bi-level positive airway pressure; CHS, central hypoventilation syndrome; CNS, central nervous system; CPAP, continuous positive airway pressure; GRADE, Grading of Recommendations Assessment, Development and Evaluation; LTM, laryngo-tracheomalacia; NIV, non-invasive ventilation; NMD, neuromuscular disorder; OSA, obstructive sleep apnea; PRISMA, Preferred Reporting Items for Systematic Reviews and Meta-Analyses; PRS, Pierre Robin sequence; ROBINS-I, Risk of Bias in Non-Randomized Studies of Interventions; SIDS, sudden infant death syndrome; SMA1, spinal muscular atrophy type 1.
